# Presenters at chiropractic research conferences 2010–2019: is there a gender equity problem?

**DOI:** 10.1186/s12998-023-00498-w

**Published:** 2023-08-10

**Authors:** Sasha L Aspinall, Casper Glissmann Nim, Steen Harsted, Amy Miller, Cecilie K Øverås, Eric J Roseen, James J Young, Karen Søgaard, Greg Kawchuk, Jan Hartvigsen

**Affiliations:** 1 School of Allied Health, Murdoch University, Perth, Australia; 2 Medical Research Unit, Spine Centre of Southern Denmark, University Hospital of Southern Denmark, Middelfart, Denmark; 3 Department of Regional Health Research, University of Southern Denmark, Odense, Denmark; 4 Center for Muscle and Joint Health, Department of Sport Science and Clinical Biomechanics, University of Southern Denmark, Odense, Denmark; 5 School of Chiropractic, AECC University College, Bournemouth, UK; 6 Department of Public Health and Nursing, Norwegian University of Science and Technology (NTNU), Trondheim, Norway; 7 Section of General Internal Medicine, Department of Medicine, Boston University Chobanian & Avedision School of Medicine and Boston Medical Center, Boston, MA USA; 8 Schroeder Arthritis Institute, Krembil Research Institute, University Health Network, Toronto, Canada; 9 Department of Clinical Research, University of Southern Denmark, Odense, Denmark; 10 Department of Physical Therapy, Faculty of Rehabilitation Research, University of Alberta, Edmonton, AB Canada; 11 Chiropractic Knowledge Hub, Odense, Denmark

**Keywords:** Chiropractic, Academia, Gender, Diversity, Disparity, Equity

## Abstract

**Background:**

Presenting at professional and scientific conferences can be an important part of an individual’s career advancement, especially for researchers communicating scientific findings, and can signal expertise and leadership. Generally, women presenting at conferences are underrepresented in various science disciplines. We aimed to evaluate the gender of presenters at research-oriented chiropractic conferences from 2010 to 2019.

**Methods:**

We investigated the gender of presenters at conferences hosted by chiropractic organisations from 2010 to 2019 that utilised an abstract submission process. Gender classification was performed by two independent reviewers. The gender distribution of presenters over the ten-year period was analysed with linear regression. The association of conference factors with the gender distribution of presenters was also assessed with linear regression, including the gender of organising committees and abstract peer reviewers, and the geographic region where the conference was hosted.

**Results:**

From 39 conferences, we identified 4,340 unique presentations. Women gave 1,528 (35%) of the presentations. No presenters were classified as gender diverse. Overall, the proportion of women presenters was 30% in 2010 and 42% in 2019, with linear regression demonstrating a 1% increase in women presenting per year (95% CI = 0.4–1.6%). Invited/keynote speakers had the lowest proportion of women (21%) and the most stagnant trajectory over time. The gender of conference organisers and abstract peer reviewers were not significantly associated with the gender of presenters. Oceanic conferences had a lower proportion of women presenting compared to North America (27% vs. 36%).

**Conclusions:**

Overall, women gave approximately one-third of presentations at the included conferences, which gradually increased from 2010 to 2019. However, the disparity widens for the most prestigious class of keynote/invited presenters. We make several recommendations to support the goal of gender equity, including monitoring and reporting on gender diversity at future conferences.

**Supplementary Information:**

The online version contains supplementary material available at 10.1186/s12998-023-00498-w.

## Background

Presenting at academic conferences allows for opportunities to disseminate knowledge and research, network and exchange ideas, develop collaborations, and be eligible for awards or other recognitions. In addition, invited presentations are generally regarded as an acknowledgment of expertise and leadership within a field. Thus, these opportunities help individuals cultivate a national or international reputation which can be important for promotion and career advancement [1]. Inequities among men and women in opportunities to present at conferences are well demonstrated in academic medicine and other scientific fields [2–5]. In addition, the gender composition of organising committees can affect the gender of presenters at scientific conferences [2, 3]. The gender distribution at conferences hosted by chiropractic organisations has not been evaluated.

Gender disparities can be defined as differences between gender groups that are considered unjust or inequitable, which can include access to resources and status [6]. If women or gender minorities are underrepresented as presenters at conferences, this may have a tangible impact on their career progression that is avoidable and unjust. Moreover, visibility of women and gender minorities in research and at conferences may help to increase the rate at which members of those groups choose to enter the research workforce and pursue leadership opportunities. Ultimately, the gender distribution of presentations at chiropractic conferences should approximate the overall gender distribution of the chiropractic profession and the country or region, the latter being near 50% women [7]. While a gender disparity has been identified in leadership positions within the chiropractic profession [8], we are unaware of any previous investigations of gender diversity in the chiropractic scientific community.

In the chiropractic profession at large, the proportion of women chiropractors is approximately 32% in the USA [9], 40% in Norway (K Fossum-Piene, personal communication, 2022 Aug 26), 42% in Australia [10], and 45% in Canada. The UK [11] and Denmark [12] appear to be unique with 50% and 60% women chiropractors respectively. These proportions have had an upward trajectory over the prior decade in most countries [9, 12–14] except the UK [15], suggesting the chiropractic profession is headed toward gender parity.

### Aims

The overall aim of this paper was to evaluate the gender distribution of presenters at conferences with an abstract submission process hosted by chiropractic organisations from 2010 to 2019. Specifically, we aimed to evaluate if gender distribution changed between 2010 and 2019. Second, we aimed to determine if conference characteristics were associated with the gender distribution of presenters, including the gender distribution of the organising committee, gender distribution of peer reviewers, and the geographic location of the conference.

## Methods

This was a retrospective study in which we investigated the gender of presenters at conferences with an abstract submission process hosted by five chiropractic organisations from 2010 to 2019. Ethics approval was acquired from the Murdoch University Human Research Ethics Committee (approval 2022/007).

### Conference selection criteria

We included chiropractic conferences that accepted research abstracts (podium and/or poster presentations) and were hosted by chiropractic organisations in the years 2010–2019. We chose this ten-year period due to the significant COVID-related disruptions to normal conference activities that occurred in 2020 to 2022. The decision to only include conferences with abstract submission was to enable a focus on researchers within the profession, rather than on conferences more specifically aimed at clinicians. Conferences were not included if we were unable to retrieve any data on the conference presenters.

Therefore, conferences hosted by the World Federation of Chiropractic (WFC), Association of Chiropractic Colleges (ACC), European Chiropractors’ Union (ECU), Australian Chiropractors Association (AusCA, formerly Chiropractor’s Association of Australia), and Chiropractic Australia (CAus, formerly Chiropractic and Osteopathic College of Australasia) were eligible.

### Data collection

First, we collected data on the conferences and their presenters from online proceedings and conference programs. When these were not available, the relevant organisation was contacted by email to request the information. Finally, members of the research team checked their personal email and physical records for conference program information for any remaining data that was not provided.

To pre-test data availability and data collection methods, we accessed one conference program in March 2022 (ECU Convention 2019) and extracted the relevant data, making amendments to the data collection process as needed.

#### Conference data

For each conference, data were collected on the associated organisation/s, year, location, and whether abstract submissions were reviewed for inclusion blinded to the authors. In addition, we collected the names of members of conference organising committees and abstract peer reviewers, and their qualifications, titles, and affiliations (if presented in conference materials).

#### Presentations data

For each presentation at each conference, the following data were collected:

- Name of presenter (including abstract presenting authors).
- Presenter qualifications, titles, and affiliations.
- Presentation type (categorised as keynote/invited, workshop, panel, podium abstract, poster abstract, or other).

Each presentation was treated as unique, i.e., if a single individual presented multiple times at a conference, each presentation was recorded separately. If a session had multiple presenters (e.g., a panel discussion), each person was recorded as a separate presentation. Commercial presenters, conference ‘housekeeping’ or award presenters, and session moderators were not included.

### Gender classification process

Duplicates of individuals across all conferences (presenters, organisers, and peer reviewers) were removed before each individual’s gender was evaluated. Gender was evaluated based first on title or pronoun usage in biographical information available in the conference programs. In the absence of this information, online information about the individual (e.g., institutional biographical webpages) were evaluated for pronoun or gendered title usage. A similar approach has been used previously [4]. Failing this, we evaluated names and available photographs. Final gender classification was based on agreement by two investigators independently, with discussion in the case of disagreements. If agreement was not reached, the individual would be contacted directly via a publicly available email address to ask for gender information. If detailed gender identity information was known or provided to us, this would be recorded (e.g., transgender woman).

This approach assumed each individual’s gender at the time of the presentation, or at the time of data collection, and would not distinguish individuals whose gender aligns with their sex assigned at birth (cisgender) from those whose gender does not align with sex assigned at birth (transgender). While we respect that gender is viewed as a non-binary construct, we anticipated that our method of determining gender would likely limit gender categories to gender neutral or non-binary (they/them), woman (she/her), and man (he/him). The terms “woman/man” were used as they are generally preferred over “female/male” when referring to gender identity [16].

### Statistical analysis

For reporting and analysis, we intended to collapse gender into the groups “woman,” “man,” and “gender diverse.” The gender diverse group was intended to represent any individuals who did not present as “woman” or “man”, i.e., used non-binary pronouns such as they/them. However, no individuals were classified as gender diverse, therefore this group was disregarded in all analyses. All gender data is presented in aggregated form to maintain anonymity.

The following descriptive data are reported:

- Availability of conference data.
- Gender distribution of presentations in total, and by presentation type, year, and conference.
- Gender distribution of organising committee and abstract peer reviewers in total.

We performed statistical analyses with R (v. 4.2.2) [17] using the Tidyverse language [18]. Factors associated with the gender of presenters were analysed using linear regression. All gender variables were analysed as the *Percent of women* within the relevant category (e.g., percent of women presenters from among all presenters). We ran four models as below (details in Additional File 1). Models One through Three were based on our pre-defined research questions, while model Four was added post hoc given the exploratory nature of this study and the availability of data.

1. *Percent women presenters* by *Year (overall and per presentation type)*.
2. *Percent women presenters* by *Percent women organisers* interacting with *Presentation type* (collapsed into invited and non-invited).
3. *Percent women abstract presenters* by *Percent women abstract reviewers*.
4. *Percent women presenters* by *Global region*.

The global regions used in the fourth model were North America, Europe, and Oceania (Australia). Only one conference was held in Africa and South America each, and none in Asia, hence these regions were omitted from the analysis.

## Results

### Conference inclusion and data availability

Forty conferences were eligible from the years 2010 to 2019. No records could be found for one eligible conference (CAus Conference 2011) hence it was not included. Partial or complete data were available for the remaining 39 conferences. See Table 1 for a summary of data availability, and Additional File 2 for detailed data. Complete information (names of all presenters, peer reviewers, and organisers, and peer reviewer blinding status) was available or provided for 12 (31%) conferences. The remaining conferences had various missing data. In some cases, the abstract presenters were not indicated in the available conference material, in which case the first author’s name was extracted as a substitute (recorded as partially available in Table 1).

**Table 1:** Summary of data availability for the 39 included conferences

Data	Fully available, n (%)	Partially available, n (%)	Not available, n (%)
**Speakers (excluding abstracts)**	30 (77%)	6 (15%)	3 (8%)
**Abstract presenters**	21 (54%)	11 (28%)	7 (18%)
**Peer reviewers**	33 (85%)	0 (0%)	6 (15%)
**Peer review blinding status**	29 (74%)	0 (0%)	10 (26%)
**Conference organisers**	16 (41%)	0 (0%)	23 (59%)

### Gender classification process

During gender classification, there were a total of 1,760 unique individuals (among presenters, organisers, and peer reviewers). Of these, 206 were classified based on titles or pronouns in conference material, 333 based on titles or pronouns in online biographical material, 1,214 based on name and/or photo, and 9 were unable to be classified based on the above. Of those we were unable to classify, none had publicly available email addresses thus we did not contact any individuals to ask their gender identity. There were 45 (3%) disagreements during gender classification, which were resolved by consensus. We did not classify any individuals as gender diverse as we found no explicit conference or online biographical materials using ‘they/them’ pronouns or stating a diverse gender.

### Gender of organisers and abstract peer review

There were 105 unique members of organising committees (35 [33%] women, 70 [67%] men, 0 unknown), and 2,130 unique abstract peer reviewers (716 [34%] women, 1,413 [66%] men, 1 [0.05%] unknown). Among the included conferences, 27 (69%) reported that abstract reviewers were blinded to abstract authors, while 2 (5%) were not blinded, and 10 (26%) were unknown.

### Gender of presenters

**Fig. 1:**
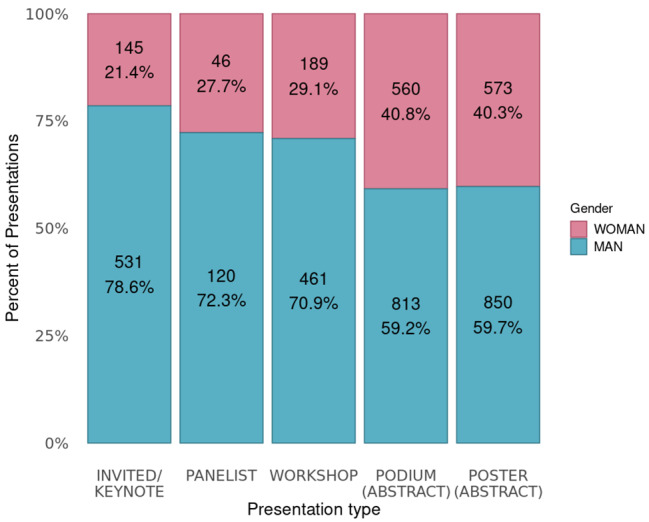
Percent of presentations from all years (2010–2019) by women and men for each presentation type Data in each column presented as n and percent. A small number of additional presentations (n = 42) were categorised as “Other” (called Innovation presentations at WFC 2016 and 2018) and are not presented in this figure

There was a total of 4,340 presentations, of which 1,528 (35%) were presented by a woman, 2,802 (65%) by a man, and 10 (< 1%) of unknown gender. By presentation type, women accounted for 21% of invited/keynote presentations, 28% of panellists, 29% of workshops, 41% of podiums, and 40% of posters (Fig. 1). There was a total of 1,577 individual presenters (regardless of how many presentations each delivered); 606 (38%) were women, 962 (61%) were men, and 9 (< 1%) were unknown.

**Fig. 2:**
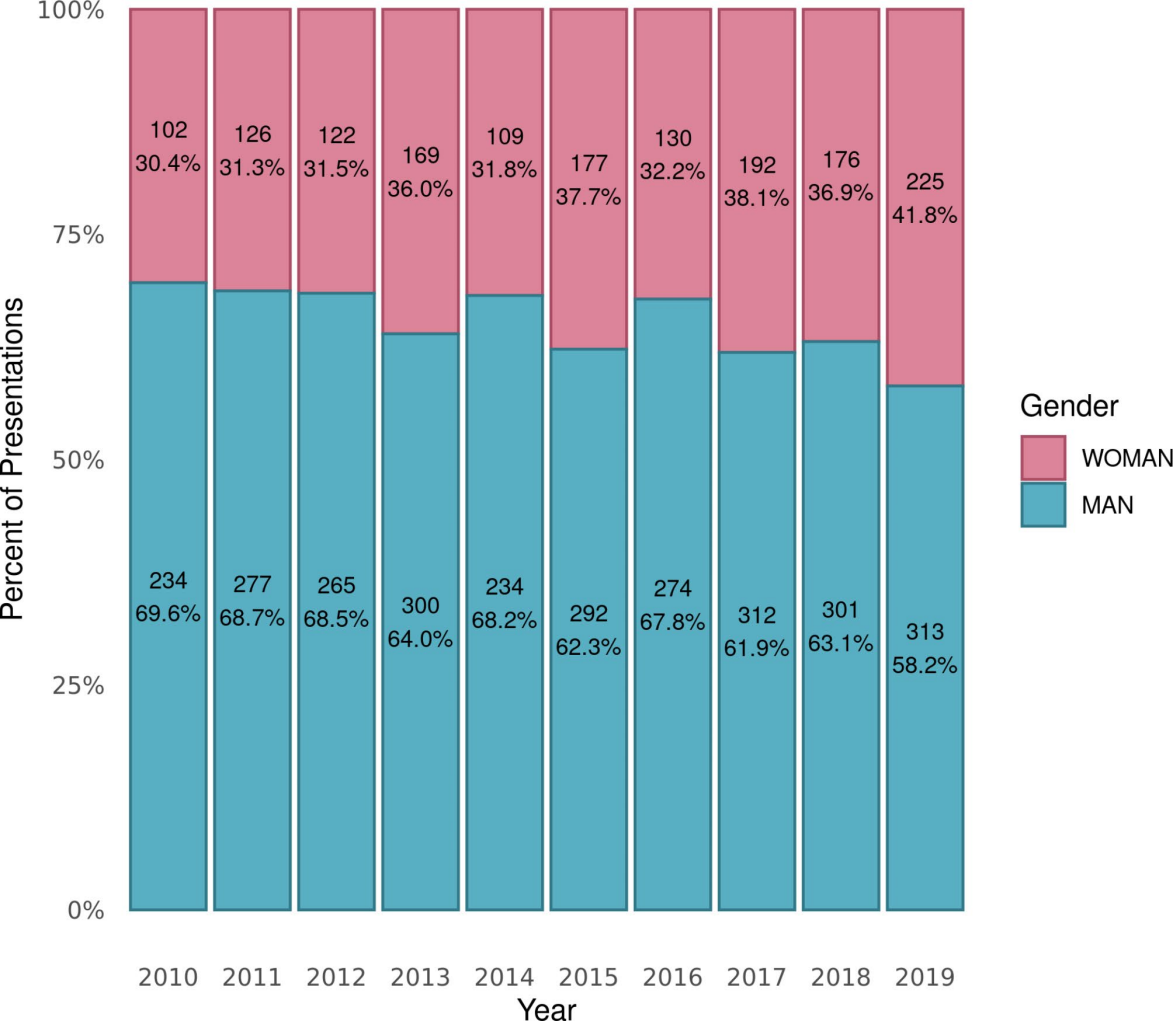
Percent of presentations by women and men per year Data in each column presented as n and percent

**Fig. 3:**
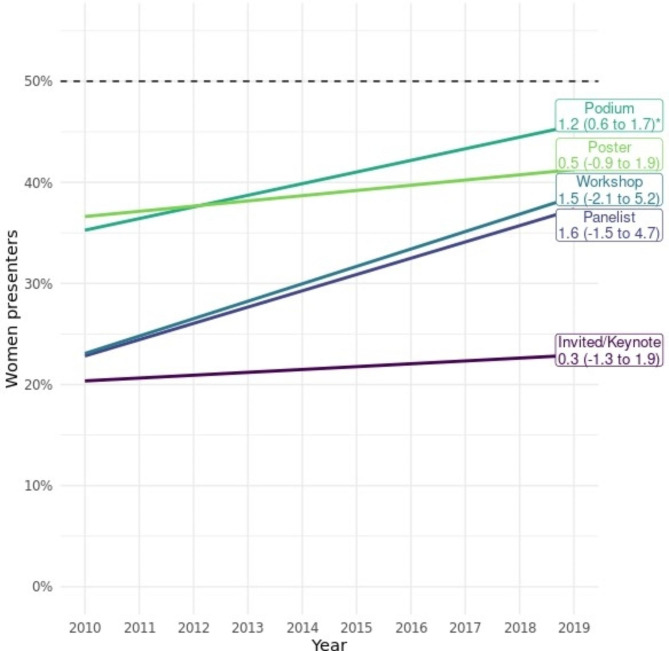
Change in the percent of women presenting by year and presentation type Data for each presentation type given as change in percent per year (95% CI). * p < .05

The overall percent of women presenters was 30% in 2010 and 42% in 2019 (Fig. 2). Linear regression demonstrated a statistically significant increase of 1% per year (95% CI = 0.4–1.7%, *p* = .004). Stratified by presentation type, podium presenters increased significantly by approximately 1% (*p* = .001) per year while the remaining presentation types did not change significantly (Fig. 3). Invited/keynote presentations had both the lowest proportion of women and the smallest average increase per year. For additional detail, see Additional File 3 for figures showing the proportion of women for each presentation type per year, and the proportion of women presenters for each conference organiser per year.

### Factors associated with presenter gender

There was no significant association between the percent of women organisers and the percent of women presenters in invited roles (β = -0.2%, 95% CI = -0.3–0.0%, *p* = .05). There was also no significant association between the percent of women abstract presenters and percent of women peer-reviewers (β = 0.1%, 95% CI = -0.1–0.3%, *p* = .30).

To explore the effect of the conference location on the gender distribution of presenters, conferences were grouped by global region; North America (n = 12), Europe (n = 12), and Oceania (n = 13). Other global regions are not included as they had one or no conferences. North America had the highest mean of women presenters (36%, 95% CI = 34–38%), followed by Europe (31%, 95% CI = 25–37%), then Oceania (27%, 95% CI = 21–33%).

## Discussion

This analysis of gender equity at scientific conferences hosted by chiropractic organisations between 2010 and 2019 showed that women accounted for 33–35% of presenters, conference organisers, and abstract peer reviewers. There was, however, a clear disparity between types of presentations; women presenters were more common for podium and poster abstracts (41% and 40% respectively), while only 21% of invited/keynote presentations were given by women.

There was an overall increase in the proportion of women presenters of approximately 1% per year between 2010 and 2019. This appears to be primarily explained by a progressive increase in women presenting podium abstracts, from 37% to 2010 to 49% in 2019. Conversely, women as invited/keynote presenters had the most stagnant trajectory across the same period.

The gender of organisers and of abstract reviewers did not have a significant impact on the proportion of women presenters at the conferences. Conferences held in Oceania had a significantly lower proportion of women presenters compared to North America, with European conferences falling between the two.

The overall proportions of women presenting in 2018 and 2019 (37% and 42% respectively) are similar to recently reported proportions of women chiropractors in the USA and Australia (32% and 42% respectively) [9, 10] but are clearly lower than the proportion of women in the profession in the UK and Denmark (50% and 60% respectively) [11, 12]. Hence, it is apparent that the gender of presenters is not always representative of the gender of the profession. Our observations mirror and slightly exceed the findings at conferences among various medical disciplines, where women tend to represent around 30–33% of speakers [2, 19, 20], sometimes reflecting the demographics of those within the same field and sometimes with women underrepresented [2].

Our data tells a similar story to a survey of people in leadership positions (academic, professional, and regulatory organisations) in the chiropractic profession in Canada and the USA [8]. They found that 31% of leaders identified as women, and that this disparity widened in ‘principal’ leadership positions - only 14% of which were held by women. This is similar to our observation that there was substantially higher disparity for invited/keynote speakers (often considered leaders) than for abstract presenters (often people who are relatively early career).

Together, these data demonstrate that the disparity between proportions of men and women increase at more advanced career stages in chiropractic. This trend is demonstrated in many other fields [21–24] and probably reflects that the chiropractic profession historically has been male-dominated. It is likely there is a time lag occurring between the gender of chiropractors and of those in positions of leadership and expertise within the profession. However, it is important to highlight that invited/keynote presenter gender maintained the flattest trajectory between 2010 and 2019, suggesting there may be a ‘glass ceiling’ effect involved. Possibilities include gender bias in invitations to present, as well as the so-called ‘leaky pipeline’ where women tend to leak out from career pipelines at greater rates than men for a variety of complex reasons [24, 25].

No individuals were classified as gender diverse; however, it is likely a portion of individuals in our sample would self-identify outside of the gender binary or as transgender. Estimates of the proportion of chiropractors who identify with a gender minority are scarce as many registration bodies and surveys have not collected this information. Canadian [26] and USA [9] data suggest around 0.2% of chiropractors are gender diverse. In our data set, this would equate to roughly nine individuals. It is also possible that people from gender minorities are underrepresented in science and leadership positions in the profession, as they are known to face additional barriers in the workplace [27, 28].

It has been found in several disciplines that better gender equity on conference organising committees tends to result in improved gender representation among speakers [2, 3, 29]. This relationship was not observed in our analysis; however, this may be because we lacked data on conference organisers for most conferences (59%). Thus, chiropractic conference organisers should still be mindful of the potential that more diverse organising committees may result in more diverse presenters. Others have highlighted that deliberate actions by conference organisers are often required to create positive change in this space [3, 29]. Also, in some organisations it is recognised that just providing data on the gender distribution influences awareness among the organisers.

Finally, the absence of a significant association between the gender of abstract presenters and of peer reviewers is encouraging, and potentially because the majority of conferences (69%) conducted abstract peer review in a blinded manner. This would diminish the impact of potential conscious or unconscious gender bias on the decision-making process.

### Methodological considerations

We acknowledge that our method of evaluating gender is inherently limited in various ways, but most importantly because it did not allow each speaker to nominate their own gender. We respect that a person’s gender identity may change over time, and our approach was also unable to capture this. Since this study focused on chiropractic conferences that included an abstract submission process, we cannot make conclusions about multidisciplinary conferences or those aimed more specifically at chiropractic clinicians, which might have different presenter demographics.

### Recommendations

At a minimum, we encourage all chiropractic conference organisers to actively record, monitor, and publish data on diversity, and to do so using inclusive gender options. This includes among presentation types, organisers, abstract peer reviewers, and even attendees. Resources to support this type of data collection exist, including a comprehensive tool for monitoring gender at academic conferences [1], and Stonewall’s “Do Ask, Do Tell” guide [30] that discusses how to gather inclusive gender information.

To facilitate improved gender representation at chiropractic conferences, there are a range of other specific actions that could be taken. This includes:

- Statements in support of gender diversity could be included in conference material and call for abstracts.
- Organisers should explicitly consider the gender of invited and keynote speakers, especially aiming for the gender of speakers to reflect the gender of attendees and/or the profession at large.
- Organisers may wish to set criteria e.g., maximum % men and no so-called ‘manels’ (panels only consisting of men).
- Organising teams should be diverse themselves.

In the broader context of gender diversity in the chiropractic profession, ongoing recording, monitoring, and publication of this kind of data should be prioritised. This may be part of a wider diversity and inclusion monitoring programme and could be used to identify and begin to address any areas of underrepresentation.

## Conclusion

We found that women overall represented 35% of presenters at chiropractic conferences between 2010 and 2019, showing an increase of approximately 1% per year. The proportion of women presenting podium abstracts increased over the period but remained fairly stagnant around 21% for invited/keynote speakers. We recommend conference organisers take specific actions to support gender equity, beginning with monitoring and reporting on gender diversity at future events.

### Electronic supplementary material

Below is the link to the electronic supplementary material.

Supplementary Material 1

Supplementary Material 2

Supplementary Material 3

## Data Availability

Anonymised data summarising gender of presenters, organisers, and peer reviewers by conference and year available on request from the corresponding author.

## Abbreviations

ACC: Association of Chiropractic Colleges
AusCA: Australian Chiropractors Association
CAus: Chiropractic Australia
ECU: European Chiropractors’ Union
WFC: World Federation of Chiropractic
