# Supplementary material for: Presenters at chiropractic research conferences 2010–2019: is there a gender equity problem?
Source: Chiropr Man Therap. 2023 Aug 10;31:28. doi: 10.1186/s12998-023-00498-w (PMC10416520; doi:10.1186/s12998-023-00498-w)
Supplement: Supplementary file 1 — Supplementary Material 1 [file 12998_2023_498_MOESM1_ESM.pdf]

Additional File 1. Linear Regression Model Details.

Model 1: Change over Time

Simple linear regression was used to model changes in the gender of presenters over time. Model: *Percent women presenters* ~ *Year*. This was also run for each presentation type separately, excluding the "Other" presentation type which only appeared in two years and accounted for 42 presentations. These were so-called Innovation presentations at two WFC conferences.

Model 2: Effect of Organiser Gender

Multiple linear regression was used to model the effect of conference organisers gender interacting with presentation type on presenter gender. Presentation types were collapsed into "invited" (invited/keynote and panelist) and "non-invited" (everything else) in order to separate presentations that presumably arise due to a specific invitation from organisers. Model: *Percent woman presenters* ~ *Percent Women Organisers* * *Presentation type* (invited/non-invited).

Model 3: Effect of Abstract Reviewer Gender

Simple linear regression was used to model the effect of abstract peer-reviewer gender on abstract presenter gender (posters and podiums). Model: *Percent women presenters* ~ *Percent women abstract reviewers*. We had planned to investigate this relationship with the additional factor of whether the abstract peer-review process was blinded. However, blinding data was highly skewed toward being blinded (69.2%) or was unknown (25.6%), hence this factor was omitted.

Model 4: Effect of Global Region

To explore whether the location in which the conference was conducted affected the gender of presenters, we grouped conferences by the global regions of North America, Europe, and Oceania. North America included conferences held in the USA and Canada, Europe included conferences in Cyprus, Germany, Greece, Hungary, Ireland, the Netherlands, Norway, Switzerland, Spain, and the UK, and finally Oceania included conferences in Australia. Only one conference was held in Africa (South Africa) and South America (Brazil) each, and none in Asia, hence these regions were omitted. Model: *Percent women presenters* ~ *Global Region*.
