# Supplementary material for: Presenters at chiropractic research conferences 2010–2019: is there a gender equity problem?
Source: Chiropr Man Therap. 2023 Aug 10;31:28. doi: 10.1186/s12998-023-00498-w (PMC10416520; doi:10.1186/s12998-023-00498-w)
Supplement: Supplementary file 2 — Supplementary Material 2 [file 12998_2023_498_MOESM2_ESM.pdf]

# Additional File 2. Conference Data Availability.

Conferences with complete data included most conferences hosted by WFC, and three ECU Conventions. All remaining conferences had at least some missing information.

Limited information was available for the CAus conferences in particular. CAus was largely unable to provide names of organising committees or peer reviewers (due to confidentiality concerns) and did not confirm whether peer review was conducted blinded. We were unable to retrieve complete program information for most years. A numerical summary of 2018 abstract presenters' gender and 2019 peer reviewer's gender was provided to us by CAus and used in data analysis.

All ACC-RAC abstracts and peer review information were available publicly via the Journal of Chiropractic Education, however presenting authors were not indicated and hence first authors were recorded as a substitute.

**Table 2.1. Details of conference data availability.**

| Year | Conference Name | Location | Speaker names | Abstract presenter names | Peer reviewer names | Organiser names | Peer review blinding status |
|---|---|---|---|---|---|---|---|
| 2010 | ACC-RAC Conference | Las Vegas, USA | Yes | Partial* | Yes | Yes | Yes |
| 2011 | ACC-RAC Conference | Las Vegas, USA | Yes | Partial* | Yes | Yes | Yes |
| 2012 | ACC-RAC Conference | Las Vegas, USA | Yes | Partial* | Yes | No | Yes |
| 2013 | ACC-RAC Conference | Washington DC, USA | Yes | Partial* | Yes | No | Yes |
| 2014 | ACC-RAC Conference | Orlando, USA | No | Partial* | Yes | No | Yes |
| 2015 | ACC-RAC Conference | Las Vegas, USA | No | Partial* | Yes | No | Yes |
| 2016 | ACC-RAC Conference | Orlando, USA | No | Partial* | Yes | No | Yes |
| 2018 | ACC-RAC Conference | Dallas, USA | Yes | Partial* | Yes | No | Yes |
| 2019 | ACC-RAC Conference | Baltimore, USA | Yes | Partial* | Yes | No | Yes |
| 2015 | AusCA Conference | Melbourne, Australia | Yes | Yes | Yes | No | Yes |
| 2016 | AusCA Conference | Cairns, Australia | Yes | Yes | Yes | No | Yes |
| 2017 | AusCA Conference | Canberra, Australia | Partial | Yes | Yes | No | Yes |
| 2018 | AusCA Conference | Hobart, Australia | Yes | Yes | Yes | No | Yes |
| 2019 | AusCA Conference | Melbourne, Australia | Yes | Yes | Yes | No | Yes |
| 2010 | CAus Conference | Melbourne, Australia | Partial | No | No | No | No |
| 2014 | CAus Conference | Sydney, Australia | Partial | No | No | No | No |
| 2015 | CAus Conference | Melbourne, Australia | Partial | No | No | No | No |
| 2016 | CAus Conference | Sydney, Australia | Yes | No | No | No | No |
| 2017 | CAus Conference | Melbourne, Australia | Yes | No | No | No | No |
| 2018 | CAus Conference | Sydney, Australia | Yes | Gender split | No | No | No |
| 2019 | CAus Conference | Gold Coast, Australia | Partial | Most | Gender split | No | No |
| 2010 | ECU Convention | London, UK | Yes | No | Yes | No | Yes |
| 2011 | ECU Convention | Zurich, Switzerland | Most | No | Yes | No | Yes |

| | | | | | | | |
|---|---|---|---|---|---|---|---|
| 2012 | ECU Convention | Amsterdam, the Netherlands | Yes | Yes | Yes | Yes | Yes |
| 2013 | ECU Convention | Sitges, Spain | Yes | Yes | Yes | Yes | Yes |
| 2014 | ECU Convention | Dublin, Ireland | Yes | Yes | Yes | Yes | Yes |
| 2016 | ECU Convention | Oslo, Norway | Yes | Yes | Yes | Yes | No |
| 2017 | ECU Convention | Limassol, Cyprus | Partial | Partial* | Yes | No | No |
| 2018 | ECU Convention | Budapest, Hungary | Most | Yes | Yes | No | No |
| 2010 | WFC/CECE/ACC Education Conference | Madrid, Spain | Most | Yes | Yes | Yes | Yes |
| 2011 | WFC Congress | Rio de Janeiro, Brazil | Yes | Yes | Yes | Yes | Yes |
| 2012 | WFC/ACC Education Conference | Perth, Australia | Most | Yes | Yes | Yes | Yes |
| 2013 | WFC Congress | Durban, South Africa | Yes | Yes | Yes | Yes | Yes |
| 2014 | WFC/ACC Education Conference | Miami, USA | Yes | Yes | Yes | Yes | Yes |
| 2015 | WFC/ECU Congress | Athens, Greece | Yes | Yes | Yes | Yes | Yes |
| 2016 | WFC/ACC Education Conference | Montréal, Canada | Yes | Partial* | Yes | Yes | Yes |
| 2017 | WFC Congress + ACC-RAC Conference (DC 2017) | Washington DC, USA | Yes | Yes | Yes | Yes | Yes |
| 2018 | WFC/ACC Education Conference | London, UK | Yes | Yes | Yes | Yes | Yes |
| 2019 | WFC/ECU Congress (EPIC 2019) | Berlin, Germany | Yes | Yes | Yes | Yes | Yes |

*Abbreviations: ACC-RAC = Association of Chiropractic Colleges – Research Agenda Conference, AusCA = Australian Chiropractor's Association, CAus = Chiropractic Australia, ECU = European Chiropractor's Union, WFC = World Federation of Chiropractic. *Presenting author sometimes/always not indicated, first author recorded as substitute.*
