# Supplementary material for: Presenters at chiropractic research conferences 2010–2019: is there a gender equity problem?
Source: Chiropr Man Therap. 2023 Aug 10;31:28. doi: 10.1186/s12998-023-00498-w (PMC10416520; doi:10.1186/s12998-023-00498-w)
Supplement: Supplementary file 3 — Supplementary Material 3 [file 12998_2023_498_MOESM3_ESM.pdf]

Additional File 3. Figures with further detail on gender proportions.

This file contains figures depicting additional breakdowns of the gender of presenters between 2010 and 2019 based on presentation type (Figure 3.1) and conference organising association (Figure 3.2).

Figure 3.1. Gender of presenters by presentation type and year.

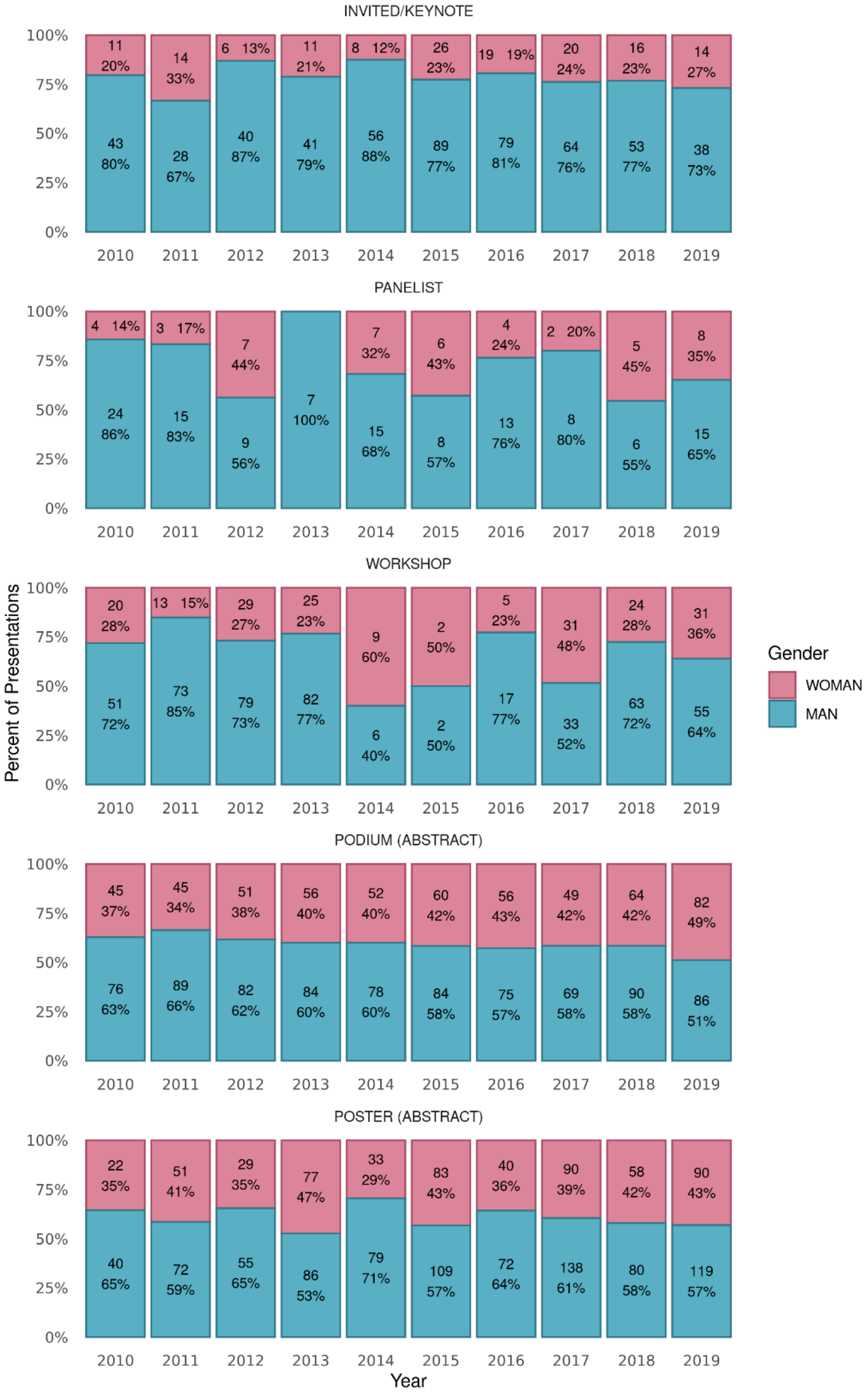

Data in each column presented as n and percent.

Figure 3.2. Gender of presenters by conference organiser and year.

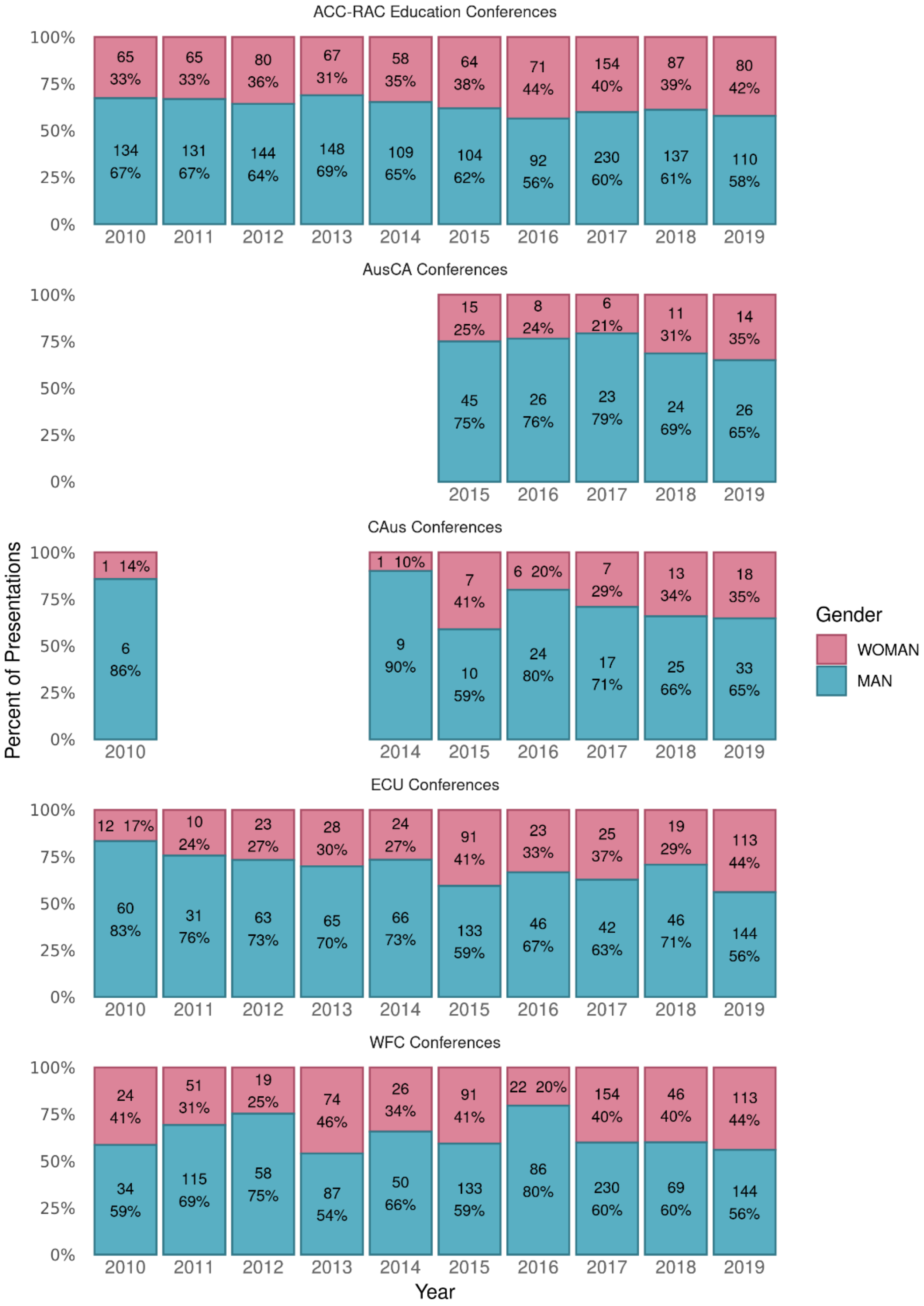

Data in each column presented as n and percent.

Abbreviations: ACC-RAC = Association of Chiropractic Colleges – Research Agenda Conference, AusCA = Australian Chiropractor's Association, CAus = Chiropractic Australia, ECU = European Chiropractor's Union, WFC = World Federation of Chiropractic.

Notes: Co-branded conferences appear under each relevant organisation in this figure (e.g., WFC/ECU Congress 2015 appears under both WFC and ECU 2015). AusCA did not include abstracts prior to 2015, hence prior conferences were ineligible. CAus did not accept abstracts in 2012 and 2013, and in 2011 we were unable to retrieve any conference data, hence these were also ineligible.
